# Role of purinergic signaling in experimental pneumococcal meningitis

**DOI:** 10.1038/srep44625

**Published:** 2017-03-16

**Authors:** Marco Zierhut, Susanne Dyckhoff, Ilias Masouris, Matthias Klein, Sven Hammerschmidt, Hans-Walter Pfister, Korcan Ayata, Marco Idzko, Uwe Koedel

**Affiliations:** 1 Department of Neurology, Klinikum Grosshadern, Ludwig-Maximilians-University, Munich, Germany; 2 Department Genetics of Microorganisms, Interfaculty Institute for Genetics and Functional Genomics, Ernst Moritz Arndt University Greifswald, Germany; 3 Department of Pneumology, Freiburg University Medical Center, Albert-Ludwigs-University, Freiburg, Germany

## Abstract

Excessive neutrophilic inflammation contributes to brain pathology and adverse outcome in pneumococcal meningitis (PM). Recently, we identified the NLRP3 inflammasome/interleukin (IL)-1β pathway as a key driver of inflammation in PM. A critical membrane receptor for NLRP3 inflammasome activation is the ATP-activated P2 purinoceptor (P2R) P2X7. Thus, we hypothesized involvement of ATP and P2Rs in PM. The functional role of ATP was investigated in a mouse meningitis model using P2R antagonists. Brain expression of P2Rs was assessed by RT-PCR. ATP levels were determined in murine CSF and cell culture experiments. Treatment with the P2R antagonists suramin or brilliant blue G did not have any impact on disease course. This lack of effect might be attributed to meningitis-associated down-regulation of brain P2R expression and/or a drop of cerebrospinal fluid (CSF) ATP, as demonstrated by RT-PCR and ATP analyses. Supplemental cell culture experiments suggest that the reduction in CSF ATP is, at least partly, due to ATP hydrolysis by ectonucleotidases of neutrophils and macrophages. In conclusion, this study suggests that ATP-P2R signaling is only of minor or even no significance in PM. This may be explained by down-regulation of P2R expression and decreased CSF ATP levels.

Bacterial meningitis is among the worldwide top ten causes of mortality from infectious diseases, and many of the survivors show significant long-term neurologic disabilities^1^. The most frequent etiologic agent in Europe and Northern America is *Streptococcus (S.) pneumoniae*, causing more than two-third of all meningitis cases^2^. Pneumococcal infection of the leptomeninges generates a powerful inflammatory reaction which contributes essentially to meningitis-associated brain damage^3^,^4^,^5^. This reaction is initiated when resident immune cells sense the presence of pneumococci by recognizing pathogen-associated molecular patterns (PAMPs) via a set of pattern recognition receptors (PRRs), namely Toll-like receptors (TLR)^5^,^6^. TLR engagement then results in myeloid differentiation primary response protein (MyD) 88-dependent production of a vast array of pro-inflammatory cytokines and chemokines^7^,^8^,^9^. As a consequence, blood-borne neutrophils are recruited in huge numbers into the leptomeninges. Activated neutrophils emit numerous cytotoxic factors which aim to kill the bacteria, but can also cause damage to neighbouring host cells.

Data from patients^10^ and animal experiments^8^,^11^ have provided evidence that excessive production of the cytokine interleukin (IL-) 1β plays a key role in the pathogenesis of PM. Consistently, we found that mice deficient in caspase-1 which converts pro-IL-1 β to its mature, biologically active form showed a strongly diminished inflammatory host response to pneumococci in the CSF^7^. We further demonstrated that lack of the inflammasome components ASC or NLRP3 that are centrally involved in caspase-1 activation also resulted in reduced brain inflammation in murine PM^9^. Moreover, using differentiated human THP-1 cells, we observed that the release of IL-β1 was timely associated with an increase in both caspase-1 activity and extracellular ATP (eATP) concentrations, and inhibition of ATP signaling by treatment with periodate-oxidized ATP attenuated IL-1β generation^9^. Accordingly, human and murine neutrophils were recently demonstrated to secrete IL-1β in a NLRP3 inflammasome-dependent manner in response to eATP and during *S. pneumoniae* corneal infection^12^.

ATP is normally present in every living cell at relatively high amounts, whereas its extracellular concentrations are considerably lower^13^,^14^. During diverse pathologic conditions, notably inflammation, ischemia, and trauma, ATP can be released from its intracellular storage pools into the extracellular space^15^. The inside-outside translocation of ATP can occur via two separate mechanisms. Inflammatory cells can secrete ATP via pannexin or connexin hemichannels^13^,^16^. In addition, ATP can be passively liberated from dying cells following nuclear and cell membrane disintegration^13^,^16^. Once in the extracellular space, ATP can behave as a “danger signal” and directly activate two families of plasma membrane receptors named P2 receptors (P2R), P2X and P2Y receptors^13^,^16^. Members of both families of P2R have been implicated in the modulation of immune responses by a variety of mechanisms including regulation of neutrophil functions, such as adherence, chemotaxis, phagocytosis, and oxidative burst, as well as macrophage function, such as non-directed migration, phagocytic clearance of apoptotic cells, and cytokine release (particularly of IL-1β)^13^,^14^,^16^. P2R signaling is terminated by diffusion of the ligand from its binding site and its subsequent phosphohydrolysis which is under the control of specific ectonucleotidases called CD39 and CD73^13^,^14^,^16^.

The role of P2R signaling in bacterial infections of the brain and its coverings, however, is widely unknown. Studies in experimental models have merely demonstrated that cortical ATP levels were decreased by about a quarter, and this loss of (intracellular) ATP was positively correlated with the extent of neuronal injury^17^,^18^. As mentioned before, recent studies proposed a role of eATP in macrophage and neutrophil response during both pneumococcal corneal infection and *in vitro*^9^,^12^. This study aimed to assess the functional relevance of eATP in pneumococcal meningitis (PM) which represents a common and very serious form of brain infection.

## Results

### P2R antagonism inhibits *S. pneumoniae* - induced macrophage activation

**Figure 1:**
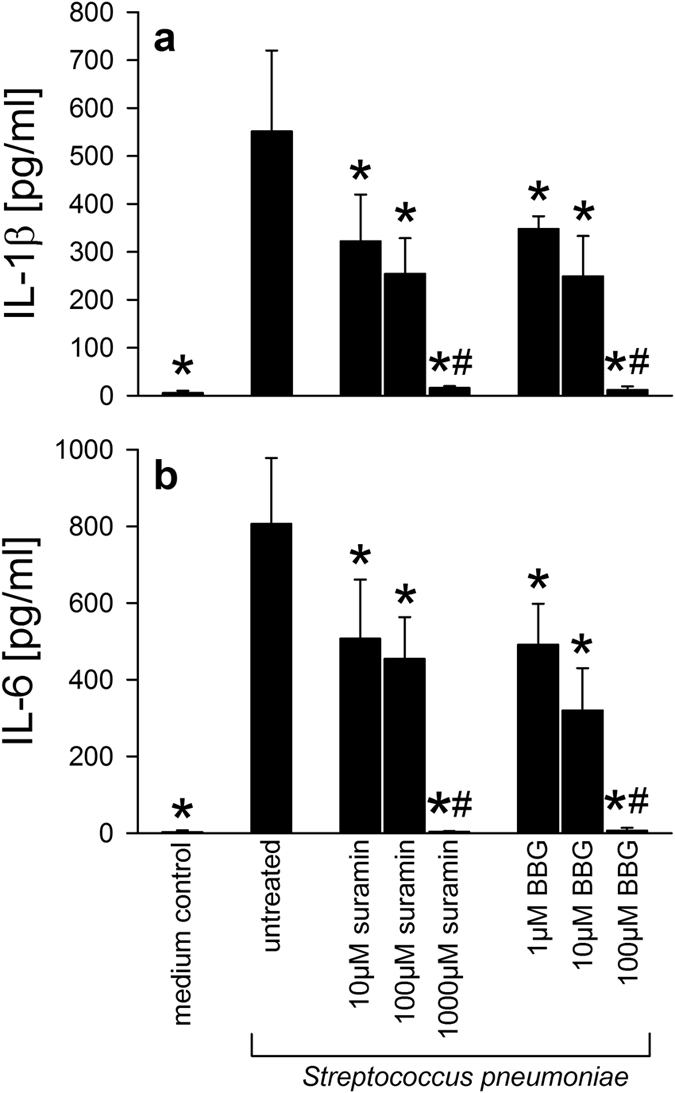
P2R antagonism modulates murine macrophage activation upon exposure to *Streptococcus pneumoniae in vitro.* To elucidate the role of P2R signaling in *S. pneumoniae*-induced macrophage activation, murine bone marrow-derived macrophages (BMDM) were exposed to D39 pneumococci (10^7 ^cfu/ml) in the absence or presence of suramin or BBG. Six hours later, medium samples were withdrawn and analysed for the presence of IL-1β (**a**) and IL-6 (**b**) using commercially available assay kits. All experiments were - at least - carried out twice in triplicates. Data are given as means ± SD. *P < 0.05, compared to *S. pneumoniae* -challenged macrophages, ^#^P < 0.05, compared to *S. pneumoniae* -challenged macrophages treated with the lower doses of the respective inhibitor, using ANOVA and Student-Newman-Keuls test for post hoc analysis.

Recent data from our group suggested an involvement of eATP in NLRP3-inflammasome–dependent IL-1β release by differentiated human THP-1 cells upon exposure to *S. pneumoniae*^19^. To confirm this hypothesis, we stimulated bone marrow-derived macrophages (BMDM) with D39 pneumococci in the absence or presence of suramin or brilliant blue G (BBG), both of which are well-known P2R receptor antagonists, albeit with significant differences in receptor subtype specificity. Suramin as well as BBG inhibited IL-1β secretion upon pneumococcal challenge in a concentration-dependent manner (Fig. 1A). Both substances also prevented pneumococci-induced production of IL-6 (Fig. 1B).

### P2R antagonism has no effect on the development of PM

**Figure 2:**
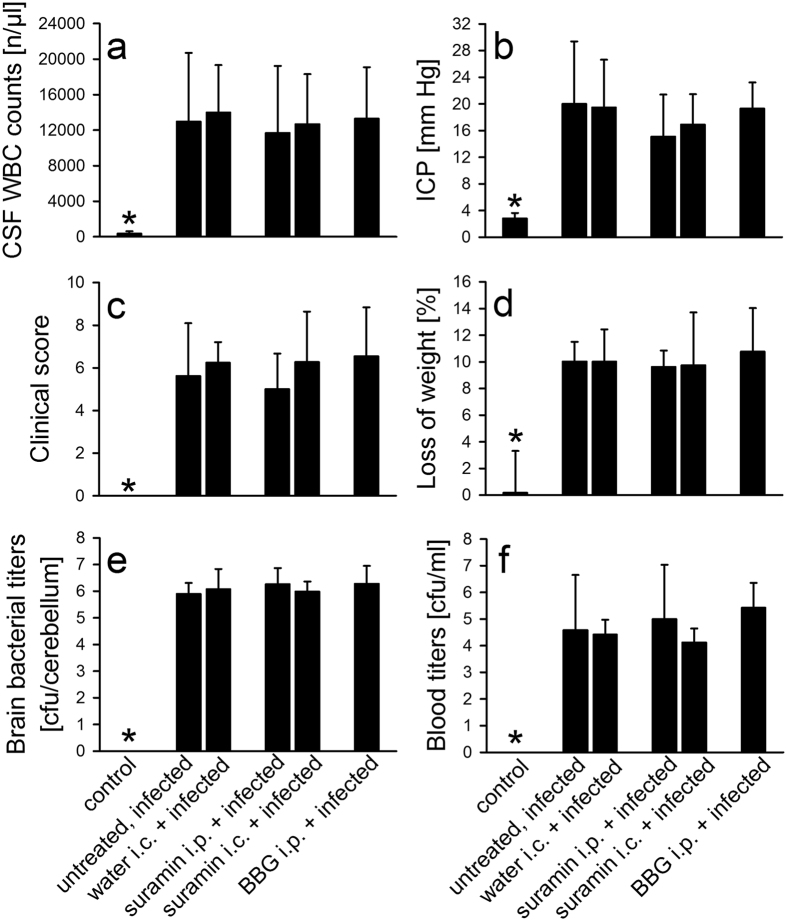
P2R antagonism has no effect on the disease course of PM. Mice were treated with suramin (given either by the intraperitoneal or the intracisternal route; 20 mg/kg body weight or brain weight, respectively; n = 11 for each group), BBG (applied intraperitoneally; 50 mg/kg body weight; n = 11), or vehicle (untreated infected mice; n = 11). In an additional experimental group, mice were injected intracisternally with water (vehicle) 2 hours prior to infection. Pneumococcal meningitis was induced by intracisternal injection of *S. pneumoniae* (strain D39). Sixteen hours later, animals were evaluated. (**a**) The number of white blood cells (WBC) in the CSF was comparable between mice that received P2R antagonists and those that were injected with vehicle. (**b**) The meningitis-induced rise in intracranial pressure (ICP) was also not altered by treatment with P2R antagonists. (**c**,**d**) There were also no between-group differences in the clinical status and in the reduction of body weight. (**e,f**) Moreover, pneumococcal outgrowth in the brain (as determined by cerebellar titres) and blood was not affected by pre-treatment with P2R antagonists. Data are given as means ± SD. *P < 0.05, compared to infected animals, using ANOVA and Student-Newman-Keuls post-hoc test.

To test the functional significance of P2R signaling in pneumococcal meningitis, mice were treated before infection with suramin or BBG, and examined 16 h later. BBG is a commonly used P2X7R antagonist capable of crossing the intact blood-brain barrier (BBB)^20^,^21^. The non-selective P2R antagonist suramin, however, was reported to penetrate only poorly into the brain under normal conditions^22^,^23^. The BBB is usually broken down in PM so the delivery of suramin across the BBB may not be an important problem. Nevertheless, in order to be sure not to miss any effect of the drug due to limited bioavailability, suramin was administered via the intracisternal (i.c.) route in an additional experimental group. Neither pre-treatment with suramin (irrespective of the application route) nor BBG had any potential impact on the development of meningitis. At 16 h after infection, the number of leukocytes in the cerebrospinal fluid (CSF) was comparable between mice that received the P2R antagonists and those that were injected with the vehicle (water, Fig. 2A). Accordingly, CSF concentrations of IL-1β were not altered by treatment with suramin or BBG (Fig. 2B). Compatible with the lack of effect on brain inflammation, there were no between-group differences in the degree of intracranial complications like the rise in ICP or the magnitude of intracerebral hemorrhages (data not shown). Correspondingly, the clinical status assessed 16 h post infection, as well as the loss in body weight (Fig. 2C,D) and temperature (data not shown) were similar between treated and control mice. In addition, treatment with suramin or BBG did not result in significant alterations of pneumococcal titres in the brain and blood (Fig. 2E,F).

### Brain P2R expression and CSF ATP levels decreased substantially during PM

**Figure 3:**
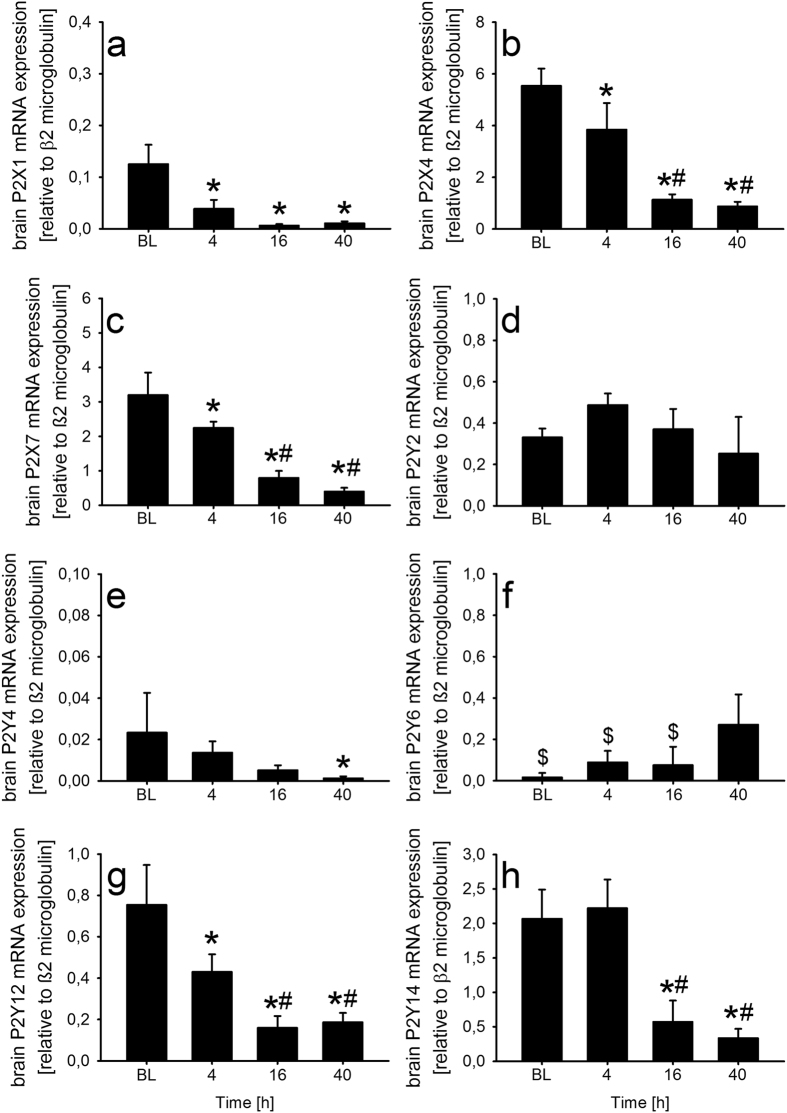
Brain P2R expression during PM. Expression of P2R subtypes in brain tissue collected from mice before, 4 hours (h), 16 h, and 40 h after the induction of PM (n = 4 each group). Relative expression of different P2 receptors (**a**–**h**) were analysed in comparison to the housekeeping gene β2 microglobulin using quantitative RT-PCR. Data are given as means ± SD. *P < 0.05, compared to uninfected controls, ^#^P < 0.05, compared to 4 h post infection, ^$^P < 0.5, compared to 40 h post infection, using unpaired Student’s *t* test and Bonferroni correction for multiple comparisons.

In order to find possible explanations for the lacking effect of P2R antagonism *in vivo*, we next investigated the expression of P2Rs in murine brains during the course of PM. As shown in Fig. 3, PM led to substantial down-regulation of most P2R subtypes including P2X1, P2X4, P2X7, P2Y4, P2Y12, and P2Y14. The expression of the P2Y2 subtype remained constant at a low level throughout the observation period, whereas a mild increase in P2Y6 mRNA expression was detectable in advanced stages of the disease.

**Figure 4:**
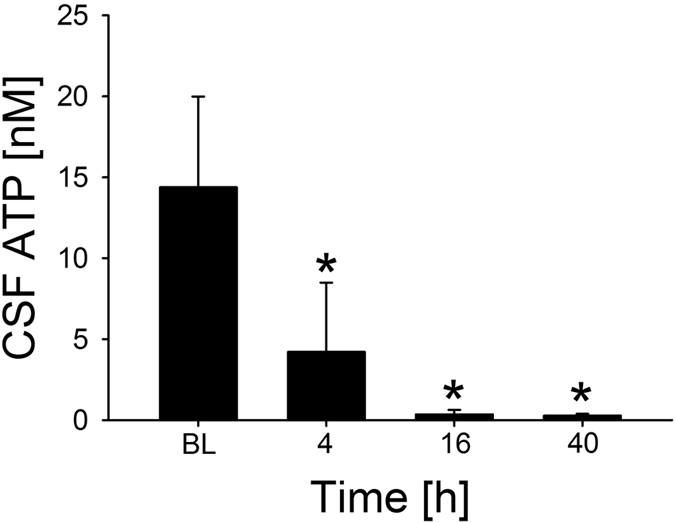
CSF ATP concentration during the course of PM. ATP concentrations were measured in CSF samples withdrawn from mice (n = 4 each group) before and at defined time points after infection with *S. pneumoniae* using a commercially available bioluminescence kit. Data are given as means ± SD. *P < 0.05, compared to PBS-injected controls (Con; n = 4), using unpaired Student’s *t* test and Bonferroni correction for multiple comparisons.

Apart from P2R expression, the extracellular nucleotide concentration is an essential determinant for the extent to which purinergic signaling can contribute to immune responses. Thus, we also measured ATP levels in murine CSF samples withdrawn before and at different time points after pneumococcal infection. CSF ATP levels markedly dropped during the course of the disease and became nearly undetectable 16 h and 40 h after pneumococcal inoculation (Fig. 4).

### Macrophage and neutrophil CD39 seems to contribute to eATP decrease

**Figure 5:**
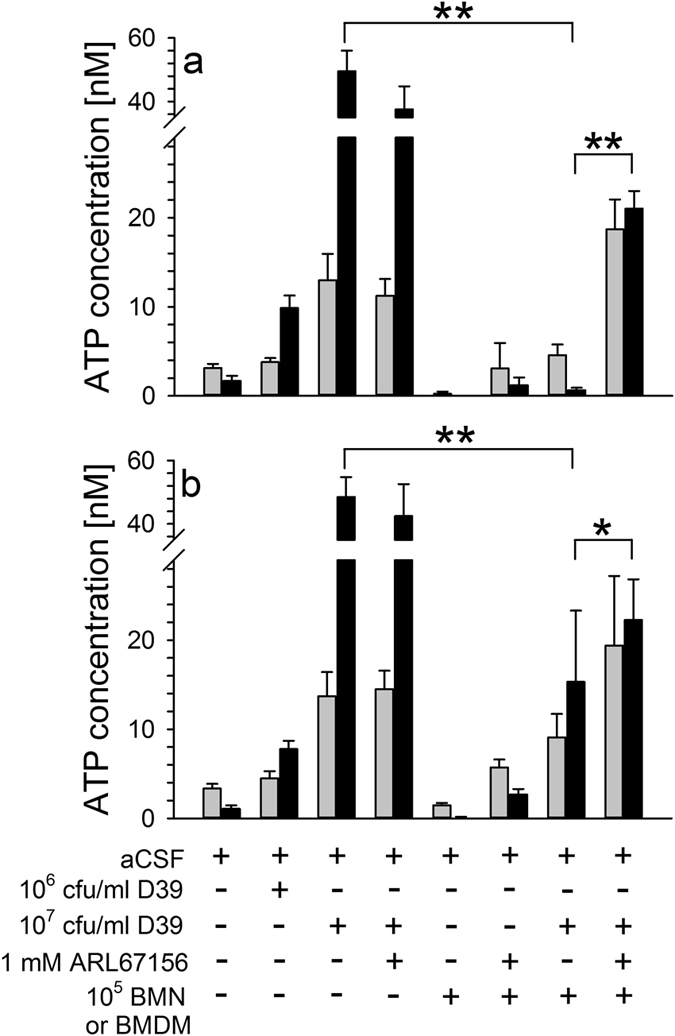
ATP catabolism by BMN and BMDM *in vitro.* *S. pneumoniae* was grown in the absence or presence of murine BMDM (**a**) or BMN (**b**) (100,000 cells per well) in artificial CSF to which ATP was added at its physiological concentration. After 30 min (grey bars) and 120 min (black bars) of culture, artificial CSF samples were taken and used for ATP determinations using a commercially available bioluminescence kit. When indicated, the ectonucleotidase inhibitor ARL67156 (1 mM) was given to the culture medium prior to the addition of *S. pneumoniae* and murine immune cells. All experiments were – at least - carried out twice in triplicates. Data are given as means ± SD. **P < 0.001 und *P < 0.01, compared to respective group, using ANOVA and Student-Newman-Keuls post-hoc test.

Since macrophages and neutrophils are major players of the inflammatory response in PM, we questioned whether these cells have a role in the observed decrease in ATP concentrations in the CSF. In order to address this aspect, we grew live *S. pneumoniae* D39 in the absence or presence of murine BMN or BMDM in aCSF substituted with 10 nM ATP (which represents the ATP concentration in normal rodent CSF^24^). After 30 min and 120 min of culture, aCSF samples were taken and used for ATP determinations. Pneumococcal outgrowth within cell-free aCSF was accompanied by a time-dependent increase in ATP levels. This increase was attenuated and even completely prevented by the presence of BMN and BMDM, respectively (Fig. 5). The ATP-degrading activity of BMN and BMDM could be – at least in part – blocked by treatment with ARL67156, hinting at a possible role of CD39 in the meningitis-associated decrease in CSF ATP.

In order to exclude that BMN and/or BMDM indirectly affect eATP concentrations by limiting pneumococcal outgrowth within the aCSF, we also determined pneumococcal titres at the end of each experiment by culturing serial dilutions of aCSF samples on agar plates. There were no differences in pneumococcal outgrowth, irrespective of whether they were grown in the absence or presence of BMN or BMDM (e.g., pneumococcal titres in the absence and presence of BMDM after 30 min of culturing, 7.14 ± 0.08 vs. 7.06 ± 0.14 log_10 _cfu/ml, and 120 min of culturing, 7.67 ± 0.07 vs. 7.71 ± 0.12 log_10 _cfu/ml, respectively; not significant).

## Discussion

Excessive neutrophilic inflammation contributes to brain pathology and consequently an unfavorable outcome of PM^3^,^4^,^6^. Among the host factors that drive inflammation are IL-1 cytokine family members. Concentrations of IL-1β are elevated in CSF samples from patients with bacterial meningitis and correlate significantly with CSF leukocyte counts and clinical outcome^10^. In animals, i.c. injection of recombinant IL-1β was sufficient to induce meningitis^25^. Moreover, pharmacologic and genetic blockade of IL-1β production or signaling attenuated meningeal inflammation and improved the clinical course of PM^7^,^8^,^9^,^26^. Recent studies demonstrated a central role of eATP in *S. pneumoniae*-induced-1L-β release by THP-1 macrophages and neutrophils *in vitro* as well as in murine corneal infection^9^,^12^. Therefore, we hypothesized that eATP may act as a modulator of inflammation and tissue damage in PM. Surprisingly, pharmacologic interference with eATP signaling did neither influence the host immune response nor the clinical course of PM in mice, although this approach proved to be efficient in preventing cytokine production and cell death in cultured BMDM upon exposure to pneumococci. Our *in vivo* findings contrast to numerous reports that have provided evidence for eATP as an autocrine and paracrine signaling molecule that trigger pro-inflammatory immune responses by activating cell surface P2R (for review see refs 16, 27 and 28). For instance, in models of acute neurodegeneration including cerebral ischemia, traumatic brain or spinal cord injury, subarachnoid hemorrhage, and seizures, pharmacologically or genetically induced ATP hydrolysis and/or P2R (above all P2X7) inhibition was shown to inhibit microglial activation, neutrophil inflammation, and cytokine expression^20^,^21^,^29^,^30^,^31^. This reduction in neuroinflammation was usually accompanied by neuroprotection and better outcome^30^,^32^,^33^,^34^. Similarly, purinergic signaling through P2R was demonstrated to have immunostimulant effects in diverse bacterial infection models. For example, local nucleotide application elevated the levels of cytokines/chemokines and neutrophils in peritoneal lavage fluid in mouse *Escherichia coli* peritonitis models^35^,^36^ (33;34). P2R deficiencies, in turn, were associated with blunted innate immune responses in experimental models of lung infection (e.g., with *Pseudomonas aeruginosa*^37^), sepsis induced by cecal ligation and puncture^38^, as well as septic encephalopathy^39^. Moreover, pharmacologic inhibition and/or genetic ablation of enzymes that degrade nucleotides like ATP dramatically enhanced inflammation in pneumococcal pneumonia^40^ (while treatment with ATP-degrading enzymes attenuated the immune response after intratracheal administration of LPS^41^ and in experimental polymicrobial sepsis^42^. These ATP driven innate immune response can both protect and harm the host, depending on whether the benefits (namely efficient bacterial clearance) outweigh the adverse consequences (namely bystander tissue injury). According to this thesis, blockade of ATP degradation led to enhanced lethality in experimental pneumococcal pneumonia by exaggerating harmful effects of inflammation^40^. Correspondingly, P2R (namely P2X7) signaling was shown to promote inflammation-induced brain injury during septic encephalopathy^39^ whereas its definite role in polymicrobial sepsis is still controversial, with some studies reporting deleterious action^38^ and others reporting the opposite^42^. Favorable effects of ATP and P2R signaling were also seen in *Escherichia coli* peritonitis^35^,^36^ and bacterial lung infections, e.g., with *Pseudomonas aeruginosa*^37^. These positive effects were related to an enhancement of bacterial clearance. Thus, the current literature indicates that eATP acts as key regulator of sterile and microbial inflammatory processes^16^,^27^,^28^. To our best knowledge, there are only a handful of papers reporting no effect of ATP and/or P2R signaling on acute neurodegenerative or infectious diseases. For example, deletion of P2X7 receptor was found neither to affect ischemic or excitotoxic neuronal cell death^43^ nor to contribute to control of pulmonary *Mycobacterium tuberculosis* infection^44^. This could be due to a publication bias favoring studies with positive results or, which is more likely, to the fact that eATP and P2R signaling plays a nearly universal role in shaping immune response to injury and infection. Thus, we considered our results to be unexpected and looked for possible explanations. It is now well established that purinergic signaling is a tightly regulated process and may depend, amongst others, on extracellular levels of nucleotides and the expression pattern of P2R and P1R^16^,^28^,^45^. We therefore analyzed brain mRNA expression levels of P2R as well as ATP concentration in CSF samples from mice with PM. PM was associated with a robust down-regulation of most P2R subtypes. In addition, CSF ATP became nearly undetectable during the course of the disease. The dramatic decline in eATP levels and P2R expression represent a likely explanation for the lack of any effect of P2R antagonism in PM and also for the striking differences in this aspect between PM and other acute neurological disorders like ischemia, brain injury or septic encephalopathy.

Recently, George and colleagues^46^ demonstrated that *E. coli* LPS can increase the extracellular catabolism of ATP into adenosine through ectonucleotidases by cultured microglial cells. In order to evaluate whether locally present immune cells contribute to the drop of CSF ATP levels in PM, we next cultured live *S. pneumoniae* in the absence or presence of murine BMN or BMDM in aCSF to which ATP was added at its physiological concentration^24^. In the absence of BMN or BMDM, pneumococcal outgrowth led to a substantial increase in eATP levels. This rise was undetectable in the presence of BMN or BMDM, but (at least in part) reversible by the co-administration of ARL67156. Thus, our findings are in line with that of George and colleagues^46^, and suggest a contribution of CD39 to the meningitis-associated decrease in CSF ATP.

Although purinergic signaling is essential for NLRP3 inflammasome-dependent IL-1β secretion from macrophages *in vitro*, is seems to be dispensable under *in vivo* conditions, namely in pneumococcal meningitis. To date, a two-signal model is proposed for NLRP3 inflammasome activation. In this model, the first signal ( = priming signal) is provided by microbial or endogenous molecules (e.g., pneumococcal PAMPs) that induce NLRP3 and pro-IL-1β expression through activation of the transcription factor NF-κB. The second signal (=activating signal) is triggered by a plethora of stimuli, such as ATP, particulate matters, or pore-forming toxins^47^,^48^,^49^. In pneumococcal meningitis, the pore-forming toxin pneumolysin may substantially contribute to NLRP3 inflammasome activation, as evidenced, for example, by significantly lower brain IL-1β concentrations in mice infected with pneumolysin-deficient pneumococci than in those infected with the respective wild type strain^9^.

In conclusion, the present study showed that P2R antagonism had no effect on inflammation, intracranial complications, and short-term outcome in PM. This may be explained by substantial down-regulation of P2R subtypes expression in the brain and loss of ATP in the CSF (presumably via an increased catabolism by BMN and BMDM).

## Materials and Methods

### Ethics statement

This study was carried out in strict accordance with the recommendations in the Guide for the Care and Use of Laboratory Animals (National Research Council, USA) and with the German Animal Protection Act. The study protocol was approved by the Committee on the Ethics of Animal Experiments of the Government of Upper Bavaria (Permit Numbers: 55.2-1-54-2531-143-12).

### Animal model of pneumococcal meningitis

A well-characterized mouse model was used in this study^9^,^50^. Briefly, adult male C57BL/6n mice were weighed and their body temperature was taken. Then, the mice were clinically examined and scored. Clinical scoring compromised [i] a beam balancing test, [ii] a postural reflex test, [iii] the presence of piloerection, seizures or reduced vigilance. In healthy animals, the score is 0 points; eleven points are attributed to terminally ill animals that have to be euthanized due to ethics within the observation period. After clinical evaluation, bacterial meningitis was induced by i.c. injection of 20 μl of 10^7^ colony forming units (cfu) per ml *S. pneumoniae* type 2 (D39 strain) under short term anesthesia with isoflurane. Controls were injected i.c. with 20 μl phosphate-buffered saline. Next, animals were allowed to wake up and food and water were supplied *ad libitum*. At the end of each experiment, animals were weighed again, scored clinically, and the temperature was taken. Then, mice were anaesthetized with ketamine/xylazine and a catheter was placed into the cisterna magna. CSF samples were withdrawn for the determination of CSF leukocyte counts and CSF IL-1β or ATP levels. Subsequently, intracranial pressure (ICP) was monitored using a pressure transducer. Then, blood samples were taken by transcardial puncture. After deep anesthesia with ketamine/xylazin, mice were perfused with 15 ml ice-cold PBS containing 10 U/ml heparin. The brains were removed and frozen immediately.

### Experimental groups

In order to evaluate the role of eATP in PM, C57BL6/n mice (Charles River GmbH, Germany) were infected and immediately thereafter treated with an intraperitoneal (i.p.) injection of 20 mg/kg body weight suramin (n = 11; an non-selective P2R antagonist from Tocris Bioscience, Germany)^23^,^51^ or 50 mg/kg body weight brilliant blue G (n = 11; BBG, a P2X7 R antagonist from Sigma Aldrich GmbH, Germany; both dissolved in sterile water)^20^,^21^, respectively. In an additional experimental group, suramin was given via the intrathecal route two hours before infection (n = 11; 20 mg/kg brain weight; the total brain weight was assumed to be 0.5 gram, according to data provided by Wahlsten *et al*.^52^.). This was done since suramin was reported to penetrate only poorly into the brain (with the exception of the circumventricular organs that lack an intact blood-brain barrier)^22^,^23^. Infected mice that were injected i.p. (n = 21) with sterile water served as positive controls, whereas mice which were i.c. injected with PBS were used as negative controls (n = 4). In order to exclude any impact of the intrathecal injection procedure on the disease course, a subgroup of mice (n = 5) was intrathecally treated with sterile water (20 μl; a volume corresponding to that of suramin) two hours prior to pneumococcal infection. In Supplemental Experiments, CSF and brain samples were collected from mice at various time points, namely before (n = 4), 4 h (n = 4), 16 h (n = 5), and 40 h (n = 4) after infection. These samples were used from determining CSF ATP concentrations as well as brain P2Rs mRNA expression levels.

### Determination of bacterial titers in blood and brain

Cerebella were dissected and homogenized in sterile saline. Blood samples and cerebellar homogenates were diluted serially in sterile saline, plated on blood agar plates, and cultured for 24 h at 37 °C with 5% CO_2_.

### Analysis of cerebral bleeding

Haemorrhagic spots were counted and the bleeding area was measured as described previously^50^.

### Real-time RT-PCR analysis of brain P2 receptors expression

Total RNA was isolated from homogenized brain tissue, using the RNeasy Mini-Kit (Qiagen, Hilden, Germany) following the manufacturer’s recommendations. Reverse transcription was performed using Stratascript reverse transcriptase (Stratagene, La Jolla, CA) and random primers (Invitrogen, Germany). Quantitative PCR was performed with Taqman Universal PCR Mastermix (Applied Biosystems, Foster City, CA) and preformulated primers and probe mixes (Assay on Demand; Applied Biosystems). PCR conditions were 2 min at 50 °C and 10 min at 95 °C, followed by 45 cycles of 15 s at 95 °C and 60 °C for 1 min using a thermal cycler (iCycler; Bio-Rad, Hercules, CA). PCR amplification of the housekeeping gene encoding β2-microglobulin was performed during each run for each sample to allow normalization between samples.

### Cell culture experiments

BMDM (from C57BL6/n mice) were prepared from bone marrow cells isolated from femurs as described previously^9^. After 7 days of differentiation with macrophage-colony stimulating factor (M-CSF), cells were detached from the culture dish by brief accutase^®^ treatment, washed, suspended in macrophage medium (DMEM containing 1% fetal calf serum [FCS], 1% HEPES, and 10 μg/ml penicillin plus streptomycin, all from Sigma-Aldrich GmbH), counted, and seeded at a concentration of 200,000 cells per well in a 96 well plate. BMDM were primed with 100 nM phorbol 12-myristate 13-acetate (PMA) for 24 hours. Then, the medium was replaced by macrophage medium, and cells were exposed to *S. pneumoniae* (D39 strain; at a concentration of 10^7 ^cfu/ml) for six hours in the absence or presence of the following compounds: suramin (10, 100, 1000 μM) and BBG (1, 10, 100 μM). At the end of each experiment, cell culture supernatants were collected for the determination of LDH, IL-1β, and IL-6.

In Supplemental Experiments, BMDM were suspended in artificial CSF (aCSF)^53^ (containing 1% FCS, 0.1 g/l D-lactate, 0.75 g/dl D-glucose, and 10 nM ATP), and seeded at concentration of 100,000 cells per well in a 96 well plate. Subsequently, cells were exposed to live *S. pneumoniae* (D39 strain, at a concentration either of 10^6 ^cfu/ml or 10^7 ^cfu/ml) for 30 and 120 min in the absence or presence of 1 mM ARL67156. Thereafter, cell culture supernatants were sampled for ATP determinations. In additional experiments, bone marrow-derived neutrophils (BMN) were used instead of BMDM. BMN were isolated as described previously^54^.

### Measurement of IL-1β, IL-6, and ATP concentrations

IL-1β and IL-6 concentrations in cell culture supernatants and/or CSF samples were determined by ELISA (R&D Systems, Germany), according to the manufacturer’s instructions. ATP was indirectly measured with a bioluminescence assay kit (Molecular Probes, Oregon, USA).

### Statistical analysis

The principal statistical test was one way analysis of variance and subsequent Student-Newman-Keuls post-hoc tests. Differences were considered significant at *P* < 0.05. Data are displayed as means ± SD.

## Additional Information

**How to cite this article:** Zierhut, M. *et al*. Role of purinergic signaling in experimental pneumococcal meningitis. *Sci. Rep.* **7**, 44625; doi: 10.1038/srep44625 (2017).

**Publisher's note:** Springer Nature remains neutral with regard to jurisdictional claims in published maps and institutional affiliations.
